# Cost-Effectiveness of Cardiac Rehabilitation in Patients with Coronary Artery Disease: A Meta-Analysis

**DOI:** 10.1155/2019/1840894

**Published:** 2019-06-04

**Authors:** Tomoyuki Takura, Nozomi Ebata-Kogure, Yoichi Goto, Masahiro Kohzuki, Masatoshi Nagayama, Keiko Oikawa, Teruyuki Koyama, Haruki Itoh

**Affiliations:** ^1^Graduate School of Medicine, The University of Tokyo, Tokyo 113-8655, Japan; ^2^Aichi Medical University, Aichi 480-1195, Japan; ^3^National Cerebral and Cardiovascular Center, Osaka 565-8565, Japan; ^4^Tohoku University Graduate School of Medicine, Miyagi 980-8574, Japan; ^5^Sakakibara Heart Institute, Tokyo 183-0003, Japan; ^6^Tokai University Hachioji Hospital, Tokyo 192-0032, Japan; ^7^Tokyo Metropolitan Geriatric Hospital and Institute of Gerontology, Tokyo 173-0015, Japan

## Abstract

**Background:**

Medical costs associated with cardiovascular disease are increasing considerably worldwide; therefore, an efficacious, cost-effective therapy which allows the effective use of medical resources is vital. There have been few economic evaluations of cardiac rehabilitation (CR), especially meta-analyses of medical cost versus patient outcome.

**Methods:**

The target population in this meta-analysis included convalescent and comprehensive CR patients with coronary artery disease (CAD), the status most commonly observed postmyocardial infarction (MI). Here, we evaluated medical costs, quality-adjusted life year (QALY), cost-effectiveness, mortality, and life year (LY). Regarding cost-effectiveness analysis, we analyzed medical costs per QALY, medical costs per LY, and the incremental cost-utility ratio (ICUR). We then examined the differences in effects for the 2 treatment arms (CR vs. usual care (UC)) using the risk ratio (RR) and standardized mean difference (SMD).

**Results:**

We reviewed 59 studies and identified 5 studies that matched our selection criteria. In total, 122,485 patients were included in the analysis. Meta-analysis results revealed that the CR arm significantly improved QALY (SMD: −1.78; 95% confidence interval (CI): −2.69, −0.87) compared with UC. Although medical costs tended to be higher in the CR arm compared to the UC arm (SMD: 0.02; 95% CI: −0.08, 0.13), cost/QALY was significantly improved in the CR arm compared with the UC arm (SMD: −0.31; 95% CI: −0.53, −0.09). The ICURs for the studies (4 RCTs and 1 model analysis) were as follows: −48,327.6 USD/QALY; −5,193.8 USD/QALY (dominant, CR is cheaper and more effective than UC); and 4,048.0 USD/QALY, 17,209.4 USD/QALY, and 26,888.7 USD/QALY (<50,000 USD/QALY, CR is costlier but more effective than UC), respectively. Therefore, there were 2 dominant and 3 effective results.

**Conclusions:**

While there are some limitations, primarily regarding data sources, our results suggest that CR is potentially cost-effective.

## 1. Introduction

Healthcare resources are growing increasingly sparse in advanced nations due to declining birth rates, aging populations, and weakening of the underlying economic foundations. Annual national health expenditures showed a 1.3-fold increase in the ratio of financial burden to gross domestic product (GDP) from 2015 to 2017 compared with that from 1985 to 1989 in both the Organisation for Economic Co-operation and Development (OECD) and G20 countries [1]. The continuation of public health insurance systems in advanced nations will require vigilant use of cost-effective medical technology. Coronary artery disease (CAD) has attracted considerable social attention because of its relatively high-associated medical costs.

The American Heart Association (AHA), American Association of Cardiovascular and Pulmonary Rehabilitation (AACVPR), and European Association of Cardiovascular Prevention and Rehabilitation (EACPR) [2, 3] currently recommend convalescent cardiac rehabilitation (CR) as the standard of care for patients with cardiovascular disease. CR has been proven to improve exercise capacity and quality of life (QOL) and reduce cardiovascular death and total mortality in patients with CAD [4].

Several meta-analyses have reported the beneficial clinical effects of CR by mainly investigating QOL improvements by exercise therapy, or comparisons with other interventions in patients with CAD and chronic heart failure [5, 6]. Regarding economic evaluation, cost-effectiveness of CR in different settings has been previously reported [7, 8].

To our knowledge, no previous meta-analysis has focused on patient utility values as patient-reported outcomes (PROs), in addition to cost-effectiveness analysis. While CR can be beneficial in preventing cardiovascular death and improving patients' QOL, it consumes medical resources. Therefore, the objective of this meta-analysis was to evaluate the cost-effectiveness of CR from the healthcare-payer perspective.

## 2. Methods

### 2.1. Target Technology and Population

The target population in this study was patients with CAD (mainly acute coronary syndrome (ACS) including acute MI (AMI)), who were undergoing convalescent and comprehensive CR.

CR was defined as prescribed exercises which were performed under safe conditions, with or without supervision as an inpatient, outpatient, or at home, with the goal of establishing a healthier physical condition by improving exercise capacity and reducing arteriosclerosis risk factors. Since the physical condition and degree of interest (enthusiasm for health) varies widely among patients, the exercise prescription differed depending on the purpose and patient characteristics. The exercise had 5 components: (1) type of exercise, (2) exercise intensity, (3) duration of exercise, (4) frequency of exercise, and (5) represcription due to an increase in physical activity. A CR which includes exercise therapy, patient education, and psychological or lifestyle guidance is called “comprehensive CR” [9]. The comprehensive CR in this meta-analysis was in accordance with the AHA Scientific Statement [2].

The control group of this meta-analysis included patients who were undergoing usual care (UC) with medication and lifestyle guidance only and not including those with an exercise prescription.

### 2.2. Systematic Review

We conducted an electric literature search in May 2015 (updated August 2016) and performed a comprehensive review of the literature using MEDLINE and EMBASE.

We used the keywords “cardiac rehabilitation” and “exercise training” for the parameters of rehabilitation and the keywords “cost-effectiveness,” “cost-benefit,” “cost-utility,” and “economic evaluation” for economic parameters. In general, CR is indicated when a patient's status is post-AMI or open-heart surgery and in patients with angina pectoris, great vessel disease, chronic heart failure, or peripheral arterial disease. In this meta-analysis, we targeted MI, as there are many publications with substantial evidence in this regard.

We selected randomized controlled trials (RCTs) and systematic reviews as we considered these to represent high levels of evidence. Since the number of publications was not large in this area, we incorporated a model analysis if sensitivity analysis was conducted to evaluate the robustness of the result.

We limited our search to English-language publications and searched for the period from 1990 to 2016. After titles and abstracts were reviewed, we extracted papers that compared CR with UC. Medical costs, quality-adjusted life years (QALYs), cost-effectiveness, mortality, and life year (LY) were used as the evaluation parameters in this meta-analysis. Since most study periods were <2 years, in accordance with the analysis method of the previous study, we did not perform a discount for either cost or utility. We did not exclude a study based on the number of samples.

### 2.3. Medical Costs and Treatment Efficacy

Costs associated with CR, testing, diagnosis, and treatment during the observation period, were extracted from each study (Supplementary Material Table 1). Expense items related to CR included room rent, equipment, and staff costs. Coronary angiography, echocardiography, Holter monitoring, exercise tests, electrocardiogram, blood tests, and chest X-rays were included as methods of testing and diagnosis during the observation period. We did not include patient out-of-pocket costs in this analysis (i.e., travel costs, cost for equipment purchased to participate in the CR program, and childcare cost). Healthcare system differs between countries, and we could not obtain information regarding how the CR cost was covered in each country.

We converted the unit of cost to United States Dollar (USD) using the annual average exchange rate in the published year of each study. When the study did not report the standard deviation (SD) of cost, in accordance with Furukawa's method recommended in the Cochrane Handbook, we imputed values presented in the report by Fitzgerald et al. [10]. As the SD of cost was twice the mean value in the report, we set the SD as twice the mean value.

QALY is used as an indicator of patient outcome when performing an economic evaluation. Here, we used QALY as a measure of efficacy. Rather than simply representing the extension of the survival period, QALY is obtained by weighting with the utility value that contains QOL. If QALY is used as the evaluation index, both survival (quantitative profit) and QOL (qualitative benefit) can be evaluated at the same time. Utility values are measured on a scale of 0 to 1, where 0 represents death and 1 represents perfect health (Supplementary Material Figure 1). Direct and indirect methods can be used to evaluate utility. The direct method involves asking patients to estimate their QOL value relative to their health condition, whereas the indirect method involves calculating utility values using a scoring algorithm from the answers obtained from the QOL questionnaire. The most commonly used direct methods are the standard gamble (SG) and the time-trade-off (TTO); the most common indirect methods are the EuroQol-5 dimension (EQ-5D) and Health Utilities Index (HUI) [11].

When a study did not report the SD of a utility value, in accordance with the Cochrane Handbook, we used sample size, 95% confidence interval (CI), or standard error to calculate the SD [12].

The TTO was used to measure patient utility in the study of Oldridge et al. [13]. As the SD was not reported in their study, we calculated the SD using the following formula:

(1) $$\begin{array}{c} \text{standard error}\left(\text{SE}\right)\times \sqrt{N}. \end{array}$$

The TTO was also used in the report by Yu et al. [14]. As this study included several phases, we added the values of all phases together and calculated the SD in a similar manner. The study by Briffa et al. [15] used the Utility-Based Quality of Life-Heart (UBQ-H) questionnaire [16], which includes TTO. As the SD was not included in that report, we calculated SD as follows:

(2) $$\begin{array}{c} \frac{\sqrt{N}\times \left(\text{upper limit}-\text{lower limit of }95\text{\% CI}\right)}{3.92}. \end{array}$$

Using the EuroQol-5 dimension questionnaire level 3 (EQ-5D-3L), Leggett et al. [17] calculated scores in the CR and UC arms of 9.77 and 9.70, respectively. As these were the results of model analysis, we imputed the mean and SD in the UC arm for meta-analysis using the report by Fitzgerald et al. [10]. As the difference between CR participants and controls reported by Leggett et al. was 0.07, we set the mean in the CR arm by adding the difference to the mean in the UC arm. As the SD of QALY was one-third the mean value in the report by Fitzgerald et al. [10], we set the SD of QALY in the CR arm as one-third the mean values. Hautala et al. [18] calculated CR and UC arm scores of 0.013 and −0.012, respectively, using the 15D questionnaire, a generic, comprehensive, 15-dimensional, and self-administered measure of health-related quality of life among adults that can be used both as a profile and single index score measurement [19]. As the SD of QALY was not described in the studies by Leggett et al. and Hautala et al., we set the SD of QALY of both arms as one-third the mean values, as reported by Fitzgerald et al. [10].

We used the difference from baseline as the standardized index in the meta-analysis of patient utility.

To ensure consistency with previous studies, we conducted meta-analyses of mortality and LY by using the literature which was collected as described in the systematic review section.

The definition of LY differs according to published reports [20, 21]. We defined LY as an evaluation index indicating the extension in years of life that is expected during the observation period. We calculated LY by subtracting the number of people who died from the number in each arm and dividing it by the same number in each arm. The SD of LY was calculated as 1-LY.

### 2.4. Cost-Effectiveness

As cost-utility analysis does not reveal the degree of cost reduction of CR over that of UC, we analyzed the medical costs per QALY, per LY, and the incremental cost-utility ratio (ICUR) to evaluate cost-effectiveness.

We calculated the medical cost per QALY by dividing the costs by the QALY. We assumed the SD of cost-effectiveness by applying the error propagation [22, 23] to both cost and utility. To avoid using negative values, we adjusted it to the absolute value where needed. We calculated the medical cost per LY by dividing the costs by LY. The SD for this was calculated as above.

We measured ICUR as the ratio of medical costs to the utility value, which estimates the cost per unit of the utility that was incurred by switching to a different treatment, and is represented as the difference of cost divided by the difference of utility. The formula used to calculate the ICUR is as follows:

(3) $$\begin{array}{c} \text{ICUR}=\frac{\text{cost of intervention arm }\left(\text{CR}\right)-\text{cost of control arm }\left(\text{UC}\right)}{\text{utility of intervention arm }\left(\text{CR}\right)-\text{utility of control arm }\left(\text{UC}\right)}. \end{array}$$

**Figure 1:**
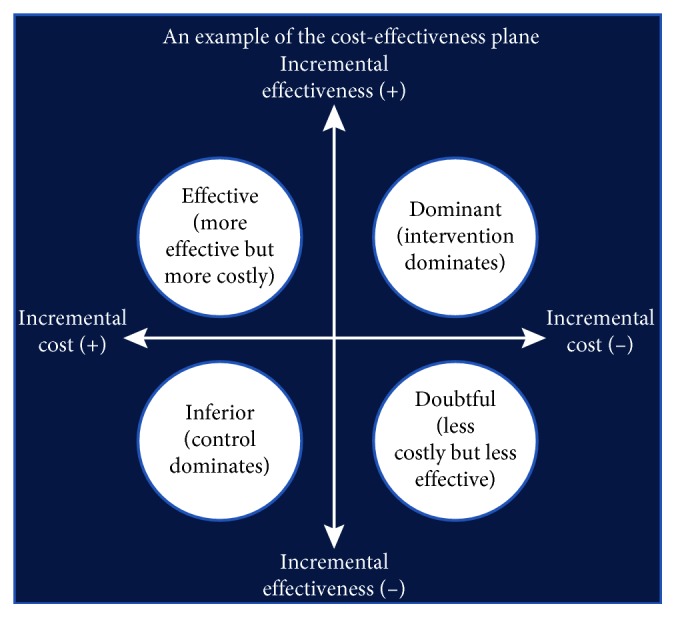
Example of the cost-effectiveness plane. Source: T. Takura, “Creating new value in medical care—methodology of social evaluation of medical technologies,” *Iyaku Keizai*, vol. 1339, pp. 16–17, 2009.

In general, the level of cost-effectiveness is expressed by the ICUR. The ICUR is compared with a predetermined threshold (a measure of decision). If this value is less than the threshold, it is categorized as cost-effective; otherwise, it is categorized as not cost-effective. When the intervention is less costly and more effective, it is categorized as dominant. When it is costlier and more effective, it is categorized as effective. If it is less costly and less effective, it is categorized as doubtful, and if it is costlier and less effective, it is categorized as dominated (Figure 1). Although there is no absolute value for the threshold of ICUR, as it varies depending on economic conditions and the perceptions of individuals, we used 50,000 USD/QALY in the United States [24–26] as a reference.

### 2.5. Meta-Analysis

We compared the differences in effects for the CR and UC categories. For dichotomous outcomes, studies were combined using risk ratios (RRs) with the corresponding 95% CIs. For continuous outcomes, standardized mean difference (SMD) with 95% CI was calculated to allow direct comparison of the results.

We used the random effects model on the grounds that there was a difference in patient population, including regional differences, for each study, and there was a possibility that the bias in each of those studies influenced the outcome of the analysis. We measured statistical heterogeneity using the *I* ^2^ statistic, and *I* ^2^ values were classified as low (<25%), moderate (<50%), or high (<75%) inconsistency [27]. A *P* value < 0.05 was considered to be statistically significant.

We conducted 1-way sensitivity analysis for QALY and 2-way sensitivity analysis for cost/QALY. We included a model analysis and assumed the mean and SD, taking into account the difference between CR and UC arms, as reported by Leggett et al. [17]. In general, if the difference does not change from the value written in the report, the meta-analysis results would not be affected by assumed values. However, as the assumed SD is not steady, we conducted one-way sensitivity analysis for QALY.

For 2-way sensitivity analysis of cost/QALY, we varied the medical cost and utility and examined the result by meta-analysis. As the number of studies used in this analysis was small, we did not consider the risk of bias.

To examine the results of cost-effective analysis, we then conducted meta-analyses of mortality and LY.

All analyses were conducted using Review Manager (RevMan for Windows, Version 5.3 Copenhagen: The Nordic Cochrane Centre, the Cochrane Collaboration, 2014).

## 3. Results

### 3.1. Systematic Review

**Figure 2:**
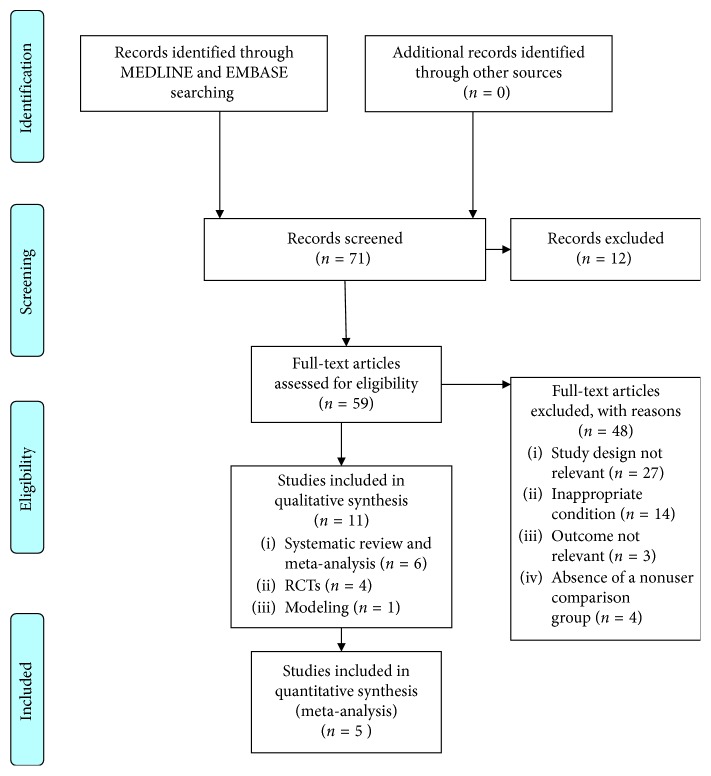
PRISMA 2009 flow diagram.

The search identified 71 potentially relevant studies. Of these, we removed 12 duplicates and excluded 48 based on the information in the titles and abstracts. Eleven articles matched the selection criteria (6 systematic reviews; 4 RCTs; and 1 model analysis). After reviewing the studies in the 6 systematic reviews, we identified 4 RCTs [13–15, 18, 28] and 1 model analysis to be included in the meta-analysis (Figure 2).

The model analysis [17] was included as it fit the criteria which were described previously, that is, those which performed cost-utility analysis to compare CR with no CR in patients who had undergone cardiac catheterization. The data source of this model analysis was the Alberta Provincial Project for Outcome Assessment in Coronary Heart Disease (APPROACH) database, which captured detailed clinical information on all patients who have undergone cardiac catheterization in Alberta since 1995 [29]. A cohort of MI or stable/unstable angina patients (*n*=121,763) captured in this database was used. Although we could not calculate LY, cost/LY, or mortality from this model analysis, we included this cohort in our meta-analysis of medical costs, QALY, and cost/QALY. Due to the publication not mentioning the numbers of patients with CR and those without CR (no CR), we referred to a previous publication [30] on the APPROACH database and defined the number of CR and no CR participants as 5,641 and 116,122, respectively, by subtracting the number of CR participants from the cohort total.

In total, there were 518 patients in the analysis of mortality, LY, and cost/LY. The analysis of medical costs, QALY, and cost/QALY included 122,485 patients. Summaries of selected studies are shown in Supplementary Material Table 2. Papers differed in reporting mortality to mean all-cause death or vascular death. Briffa et al. [15] regarded mortality to mean all-cause death. In the study by Leggett et al. [17], the duration of observation was set at 1 year because the time horizon, cost, and QALY were also calculated at 1 year.

Patient characteristics in the selected studies are shown in Supplementary Material Table 3. In the 1993 paper by Oldridge et al. [13], detailed descriptions of percutaneous coronary intervention (PCI) in the acute phase of MI, coronary artery bypass grafting (CABG), and drug therapies were not provided. Furthermore, with the exception of the report by Oldridge et al. [13], most studies did not discount medical costs and QALY, and sensitivity analyses were not addressed. Due to the nature of the analyses used, the reports by Leggett et al. [17] provided no description of patient backgrounds.

### 3.2. Meta-Analysis

**Figure 3:**
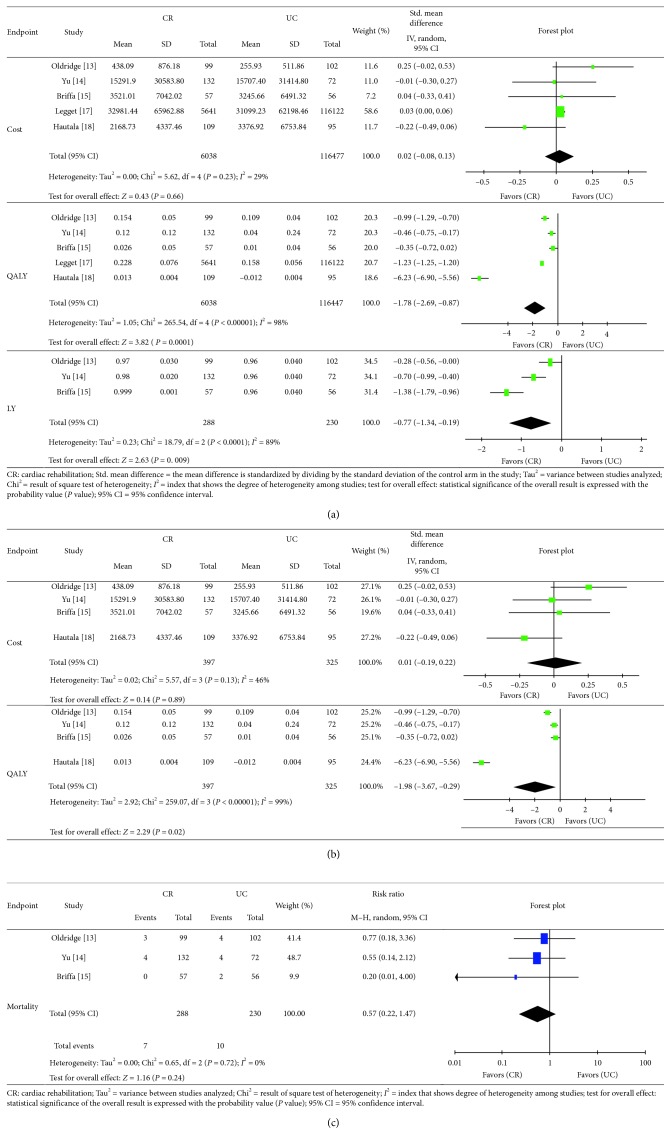
Comparison of cost, efficacy, and mortality between the cardiac rehabilitation (CR) arm and the usual care (UC) arm in patients with myocardial infarction (MI): meta-analysis. (a) Cost and efficacy, (b) cost and efficacy without the study by Leggett et al., and (c) mortality.

Although meta-analysis of medical expenses did not show a significant difference between the CR and UC arms, the CR arm had a tendency of higher expenses (SMD: 0.02; 95% CI: −0.08, 0.13). There was moderate heterogeneity among the studies (*P*=0.23, *I* ^2^=29%) (Figure 3(a)). We conducted a meta-analysis of cost without studies by Leggett et al., and it showed same tendency (SMD: 0.01; 95% CI: −0.19, 0.22) (Figure 3(b)).

Meta-analysis of QALY demonstrated that the CR arm offered a significantly better QALY than the UC arm (SMD: −1.78; 95% CI: −2.69, −0.87). There was substantial statistical heterogeneity among the studies (*P* < 0.00001; *I* ^2^=98%) (Figure 3(a)). One-way sensitivity analysis of QALY showed that changing Leggett et al.'s value of QALY while keeping the 0.07 difference between CR and UC did not affect the outcome. These results confirmed the robustness of the QALY findings. We also conducted meta-analysis of QALY without studies by Leggett et al., and it showed same tendency (SMD: −1.98; 95% CI: −3.67, −0.29) as well (Figure 3(b)).

Though we were mainly investigating cost-effectiveness, subjects in the CR arm did not show a significant difference in mortality compared to those in the UC arm. However, the CR arm had a favorable tendency of decreasing mortality (RR: 0.57; 95% CI: 0.22, 1.47). There was no evidence of significant statistical heterogeneity between the studies (*P*=0.72; *I* ^2^=0%) (Figure 3(c)).

Meta-analysis of the LY revealed that CR significantly improved LY compared with UC (SMD: −0.77; 95% CI: −1.34, −0.19). There was substantial statistical heterogeneity between the studies (*P* < 0.0001; *I* ^2^=89%) (Figure 3(a)).

**Figure 4:**
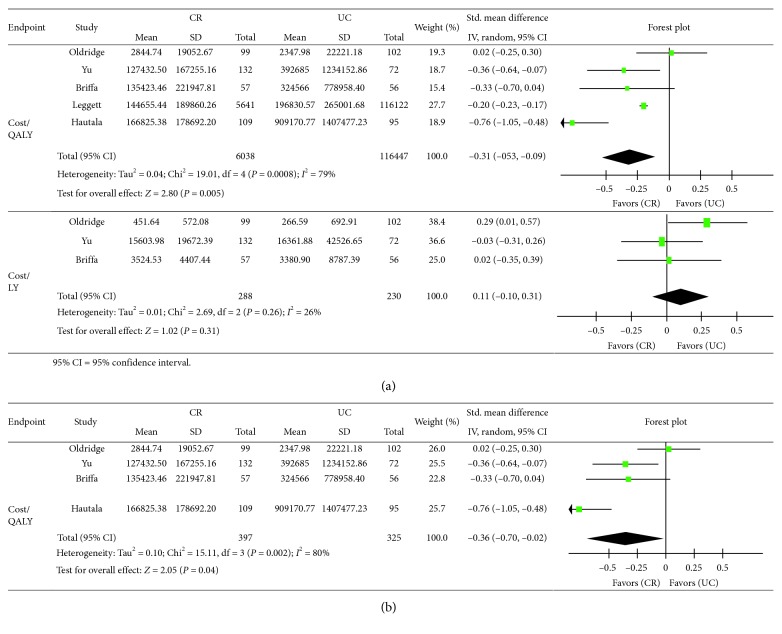
Comparison of cost-utility between the cardiac rehabilitation (CR) arm and the usual care (UC) arm in patients with myocardial infarction (MI): meta-analysis. (a) Cost-utility and (b) cost-utility without the study by Leggett et al.

Regarding cost-effectiveness, cost/QALY in the CR arm was better than that of the UC arm to a statistically significant degree (SMD: −0.31; 95% CI: −0.53, −0.09). There was substantial statistical heterogeneity between the studies (*P*=0.0008; *I* ^2^=79%) (Figure 4(a)). Two-way sensitivity analysis showed that worsening cost/QALY in the CR arm with simultaneous improvement in the UC arm of up to 11% did not affect the outcome (SMD: −0.25; 95% CI: −0.49, 0.00). In addition, we also conducted meta-analysis of cost/QALY without studies by Leggett et al. same as that above, and it showed the same tendency (SMD: −0.36; 95% CI: −0.70, −0.02) (Figure 4(b)). From these results, we conclude that the finding is robust.

The cost/LY showed no difference between the CR and UC arms (SMD: 0.11; 95% CI: −0.10, 0.31). Furthermore, there was no evidence of significant statistical heterogeneity between the studies (*P*=0.26; *I* ^2^=26%) (Figure 4(a)).

### 3.3. Evaluation of Robustness

The ICUR for each study was 4,048.0 USD/QALY (Oldridge et al. [13]), −5,193.8 USD/QALY (dominant) (Yu et al. [14]), 17,209.4 USD/QALY (Briffa et al. [15]), 26,888.7 USD/QALY (Leggett et al. [17]), and −48,327.6 USD/QALY (dominant) (Hautala et al. [18]). In summary, these results indicate 3 effective (Oldridge et al. [13], Briffa et al. [15], and Leggett et al. [17]) and 2 dominant (Yu et al. [14] and Hautala et al. [18]) results (Table 1).

## 4. Discussion

The meta-analysis indicates that CR significantly improved QALY, LY, and cost/QALY compared to UC, whereas medical cost, cost/LY, and mortality did not differ significantly between the CR and UC arms. As cost/QALY was significantly improved in patients with CR, and the ICUR for each study showed two dominant and three effective results, we suggest that CR is cost-effective when patient utility is considered. We present the results of both cost-utility analysis (CUA) and ICUR. We conducted both analyses as CUA that did not reveal the degree of cost reduction of CR over that of UC. In the studies by Briffa et al. [15] and Leggett et al. [17], the ICER was reported as 42,535 AUD/QALY (31,149.38 USD/QALY) and 37,662 CAD/QALY (27,125.31 USD/QALY), respectively. However, the range of cost differed from that of our present analysis. We calculated ICUR using cost and QALY as described earlier. In this analysis, determining cost-effectiveness using cost/LY failed to show an advantage of CR. Conversely, we observed statistically significant differences in the meta-analysis of cost-effectiveness that used QALY as the evaluation index. In addition, the ICUR results for each study were 2 dominant and 3 effective. Of the relative balance of costs and benefits, increased utility rather than increased cost was the strong tendency in CR as compared to UC. In particular, the analysis by Yu et al. [14] found that the CR arm costs more for rehabilitation, while the UC arm had more costs associated with many treatments and more frequent visits. As a characteristic of the efficacy index, large fluctuations over the short term are of greater patient utility than over the long-term survival period; thus, it is presumed that the sensitivity at the endpoint is also greatly affected.

The threshold of ICUR is a measure used to determine cost-effectiveness. In the UK, economic analysis for the evaluation of medical technology, including pharmaceuticals, has been introduced and has been used for the provision of standard treatment and prescriptions. The National Institute for Health and Care Excellence (NICE) discusses the decision-making in accordance with the position of the target drug in the public market against criteria that are judged to be capable of providing the same level as the NHS (National Health Service), as well as comparing the costs and benefits of the drug to be evaluated. In the medical technology assessment by NICE, 20,000–30,000 GBP (24,393.96–36,590.94 USD) are set as the upper limit of the cost required to gain one additional QALY. Although there are some countries that use cost-effectiveness in deciding which drugs should have costs reimbursed, the number of countries publicizing the threshold is small. In this analysis, we converted costs to USD and set the threshold at 50,000 USD. ICUR varies widely depending on the economic conditions and the perceived economic health of the country, and we should discuss the criteria for thresholds on a regional basis, depending on the circumstances of the regions where studies were conducted. However, no relevant data are available. The threshold in the United States is also not an official standard and is used as a guide. Although no ICUR thresholds have been formally established in Japan, two studies have estimated the values as 5,000,000 JPY (43,843.79 USD) and 6,700,000 JPY (58,750.68 USD), respectively, for the willingness to pay to gain 1 QALY in Japan [31, 32]. Although costs were standardized to USD, this does not fully account for differences in different regions. Exchange rates do not always ensure that a dollar has the same value in all countries. Purchasing power parity (PPP) is a common tool used by macroeconomic analysts to compare economic productivity and standards of living internationally, and we could use PPP to convert the costs. However, since PPP is based on traded goods, it might be more useful to evaluate with price indices for tradable goods, rather than nontradable price indices, as with many services [33]. Moreover, PPP is limited, in which each country's unique circumstances are not taken into consideration. Therefore, we used exchange rates to standardize the cost in this analysis.

We conducted supplementary meta-analyses of mortality and LY to examine the result of cost-effectiveness analysis. Although CR significantly improved LY compared with UC, the actual effect of CR compared with UC is still uncertain. Anderson et al. reported a meta-analysis of mortality in patients with CHD [34]. There were differences between their report and our study in the definition of CR. We defined CR as “comprehensive” CR as mentioned earlier, while Anderson et al. [34] included both comprehensive and exercise-only CR. The 2 studies did not clarify whether mortality was all-cause mortality or vascular death. Because all-cause mortality and vascular death differ, we cannot exclude their impact on the costs and LY. Moreover, as medical technology has evolved since 2005, we presume that divergence between some clinical realities and economic circumstances has occurred. With these limitations, the results of our study should be interpreted carefully.

The observation period of the included studies of mortality, LY, medical cost, and QALY ranged from 1 year [13, 15, 17, 18] to 2 years [14]. Therefore, it is necessary to consider the consistency of the observation period, and it is desirable to equalize the observation periods. For example, we should have considered using 1-year unified values. However, as it was not realistic to think that an event (death) would have occurred consistently in each of the 2 years, we did not correct values. To provide a reference point, we corrected the values in the report by Yu et al. [14] to 1 year and repeated the analysis. Consequently, the range of 95% CI became wider and the robustness decreased, but there was no change with respect to mortality. Furthermore, there was no change in the results with respect to medical costs using the 1-year analysis. These results suggest that the differences in the observation period between the studies did not have a significant impact on the present analysis. The cohort used by Leggett et al. [17] was larger than that of the other studies and had almost 99% of the overall number of patients included in this meta-analysis. While this difference might have an impact on the results, exclusion of the Leggett data showed the same tendency with respect to medical costs, QALY, and cost/QALY. Therefore, we suggest that inconsistencies in the sample size did not affect the present analysis.

Problems with meta-analysis, including integrating research with different backgrounds of participants or intervention, the risk of including low-quality studies, and the tendency not to publish negative results, have been discussed [35]. In this analysis, we have set the target disease at MI with a number of evidence. Since the number of studies used in this analysis was small, we used the paper by Yu et al. [14], in which patients with MI and PCI performed for angina pectoris were included. However, the proportion of MI patients was approximately 70% in this paper.

Regarding QALY, we should have used the effectiveness of CR itself to evaluate it accurately, but we could not separate the effectiveness of CR from medications and other variables among studies. However, as 4 of the studies used in this analysis were RCTs, we think that the bias is likely minimal. Moreover, utility values are measured on a limited scale, from 0 to 1, and small differences would not have a huge effect on the results.

Assessing the efficacy of CR in patients with CAD is not appropriate using these results because included studies were heterogeneous regarding the definition and cost of the CR and UC arms and the methods used for calculating the QALY. Currently, there are insufficient data to determine the cost-effectiveness of CR. Specifically, to evaluate the effect of CR properly, the impact on mortality and QALY must be considered over the long term (>5 years). Therefore, we support the promotion of a large-scale clinical trial to evaluate the long-term cost-effectiveness of CR.

In meta-analysis, it is important to consider not only the quality of the paper but also the selection bias. However, a funnel plot was not possible due to the small number of included studies [36]. Therefore, publication bias could not be determined.

## 5. Conclusions

This meta-analysis indicates that comprehensive CR is potentially cost-effective as determined using QALY as an evaluation index. In addition, the ICUR of each data source was dominant or effective.

## Figures and Tables

**Table 1:** Results of ICUR for comprehensive CR in patients with MI.

Study	Item	CR	UC	Difference	ICUR (USD/QALY)^*∗*^
Oldridge et al. [13]	Cost	438.09	255.93	182.16	4,048.0
	QALY	0.154	0.109	0.045	

Yu et al. [14]	Cost	15,291.9	15,707.4	−415.5	−5,193.8 (dominant)
	QALY	0.12	0.04	0.08	

Briffa et al. [15]	Cost	3,521.01	3,245.66	275.35	17,209.4
	QALY	0.026	0.01	0.016	

Leggett et al. [17]	Cost	32,981.44	31,099.23	1,882.21	26,888.7
	QALY	0.228	0.158	0.070	

Hautala et al. [18]	Cost	2,168.73	3,376.92	−1,208.19	−48,327.6 (dominant)
	QALY	0.013	−0.012	0.025	

^*∗*^ICUR rounded off to one decimal place; ICUR, incremental cost-utility ratio.

## Supplementary Materials

Supplementary Materials Supplementary Material Figure 1: concept of QALY. Recent research reports increasing use of the selection of outcome-oriented indices that apply utility (desire or satisfaction of recipients) to explain the results of medical interventions. The quality-adjusted life year (QALY) is one of the global standards used to evaluate both survival (quantitative profit) and quality of life (qualitative benefit). As a broader measure, the cost-utility analysis (CUA) is an index used to evaluate how much a health system should pay to maintain perfect health for 1 year. QALY does not necessarily cover all of the patient's health conditions; some clinical conditions have low sensitivity as a health measurement tool. Supplementary Material Table 1: medical costs and interventional behavior of selected studies by systematic review. Costs associated with CR, testing, diagnosis, and treatment during the observation period were extracted from each study. We converted the unit of cost to United States Dollar (USD) using the annual average exchange rate in the published year of each study. Supplementary Material Table 2: summaries of selected studies by systematic review. We identified 4 RCTs and 1 model analysis and summarized the details of each study, including demographics, interventions, and method of cost-effectiveness analysis. The observation period of the included studies of mortality, LY, medical cost, and QALY ranged from 1 year to 2 years. Supplementary Material Table 3: patient characteristics in selected studies by systematic review. In the 1993 paper by Oldridge et al., detailed descriptions of percutaneous coronary intervention (PCI) in the acute phase of MI, coronary artery bypass grafting (CABG), and drug therapy were not provided. The reports by Leggett et al. provided no description of patient backgrounds because it is a model analysis.
