# Supplementary material for: Cost-Effectiveness of Cardiac Rehabilitation in Patients with Coronary Artery Disease: A Meta-Analysis
Source: Cardiol Res Pract. 2019 Jun 4;2019:1840894. doi: 10.1155/2019/1840894 (PMC6589196; doi:10.1155/2019/1840894)
Supplement: Supplementary Materials — Supplementary Material Figure 1: concept of QALY. Recent research reports increasing use of the selection of outcome-oriented indices that apply utility (desire or satisfaction of recipients) to explain the results of medical interventions. The quality-adjusted life year (QALY) is one of the global standards used to evaluate both survival (quantitative profit) and quality of life (qualitative benefit). As a broader measure, the cost-utility analysis (CUA) is an index used to evaluate how much a health system should pay to maintain perfect health for 1 year. QALY does not necessarily cover all of the patient's health conditions; some clinical conditions have low sensitivity as a health measurement tool. Supplementary Material Table 1: medical costs and interventional behavior of selected studies by systematic review. Costs associated with CR, testing, diagnosis, and treatment during the observation period were extracted from each study. We converted the unit of cost to United States Dollar (USD) using the annual average exchange rate in the published year of each study. Supplementary Material Table 2: summaries of selected studies by systematic review. We identified 4 RCTs and 1 model analysis and summarized the details of each study, including demographics, interventions, and method of cost-effectiveness analysis. The observation period of the included studies of mortality, LY, medical cost, and QALY ranged from 1 year to 2 years. Supplementary Material Table 3: patient characteristics in selected studies by systematic review. In the 1993 paper by Oldridge et al., detailed descriptions of percutaneous coronary intervention (PCI) in the acute phase of MI, coronary artery bypass grafting (CABG), and drug therapy were not provided. The reports by Leggett et al. provided no description of patient backgrounds because it is a model analysis. [file 1840894.f1.docx]

**Supplementary Material Figure 1: Concept of QALY**

**
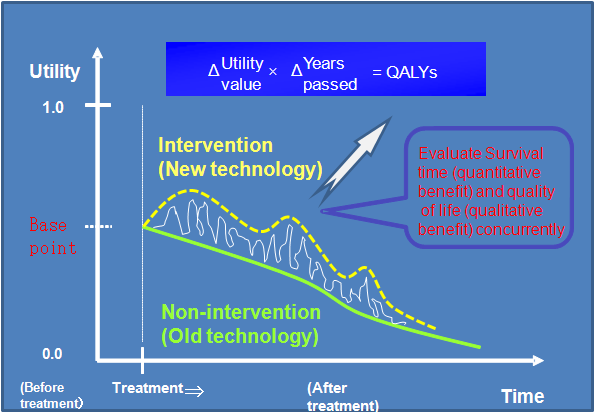
**

(Source) Takura T. Examining the current state of PCI application in Japan: from the standpoint of medical economics. Coronary Intervention. 2011 Aug;7(5):32-8

**Supplementary Material Table 1: Medical costs and interventional behavior of selected studies by systematic review**

|  | | **Cardiac rehabilitation (CR) arm** | | | | **Usual care (UC) arm** | | | |
| --- | --- | --- | --- | --- | --- | --- | --- | --- | --- |
| **Study** | **Cost  (USD)**  **Intervention** | **Cardiac rehabilitation** | **Investigations, diagnosis** | **Treatment (admission, operation, etc)** | **Total** | **Investigations, diagnosis** | **Treatment (admission, operation, etc)** | **Others** | **Total** |
|  |  |  |  |  |  |  |  |  |  |
|  |  |  |  |  |  |  |  |  |  |
| Oldridge, 1993 | Direct cost | Renting space  218.29  Equipment  48.17  Staff salaries  111.4  Community CR  60.22 | Not mentioned | Not mentioned | 438.09 | Not mentioned | Not mentioned | Community care  255.93 | 255.93 |
| Yu, 2004 | Direct cost | Staff salaries  776.6  Miscellaneous  4.6 | Coronary angiogram  871.8  Echocardiography  448.7  Holter 118.6  Exercise test  553.8  Electrocardiogram  140.4  Blood test  1013.1  Chest X-ray  48.7 | Admission 3553.6  PCI 4885  CABG 256.4  Private clinic visit　　　　 　　82  Public cardiac clinic visit  425.3  Public cardiac non clinic visit  146.3  ER 27.4  Drugs 1939.6 | 15291.9 | Coronary angiogram  1025.6  Echocardiography  448.7  Holter 118.6  Exercise test  553.8  Electrocardiogram  140.4  Blood test  1013.1  Chest X-ray  48.7 | Admission 2747.0  PCI 6481.2  CABG 205.1  Private clinic visit　　　　 53.5  Public cardiac clinic visit  435.2  Public cardiac non clinic visit  155.0  ER 30.0  Drugs 1836.5 | Staff salaries  415 | 15707.4 |
| Briffa, 2005 | Direct cost | 6-week package  508.23 | Imaging test  　1666.77  Biological test  　　 422.55 | Drugs  487.73  Consultation (ER, local doctor, heart specialist, etc)  297.32  Ambulance  138.41 | 3521.01 | Imaging test  1652.85  Biological test  385.2 | Drugs  573.41  Consultation (ER, local doctor, heart specialist, etc)  345.66  Ambulance  243.13 | Rehabilitation (details unknown)  46.14 | 3245.66 |
| Leggett,  2015 | Direct cost | Program cost  1752.32 | Not mentioned | Annual cost of care after first year  2026.01 | 32981.44 | Not mentioned | Not mentioned | Not mentioned | 31099.23 |
| Hautala, 2017 | Direct cost | Exercise-based cardiac rehabilitation costs  315.18 | Not mentioned | Primary health care costs 376.31  Secondary health care costs 1224.86  Occupational health care service costs 132.82 | 2168.73 | Not mentioned | Primary health care costs  509.13  Secondary health care costs  2613.11  Occupational health care service costs  68.52 | Not mentioned | 3376.92 |

**Supplementary Material Table 2: Summaries of selected studies by systematic review**

| Year | 1993 | 2004 | 2005 | 2015 | 2017 |
| --- | --- | --- | --- | --- | --- |
| Author | Oldridge | Yu | Briffa | Leggett | Hautala |
| Country | Canada | China | Australia | Canada | Finland |
| Objective | Effectiveness of cardiac rehabilitation in patients after acute myocardial infarction | Evaluation of long-term effects of QOL and cost-effective in the CRPP | Effects on cost and QOL by cardiac rehabilitation after acute coronary syndrome | Assess the cost utility of a center-based outpatient CR program compared with no program | Evaluation of the cost-effectiveness of exercise-based cardiac rehabilitation |
| Study design | RCT | RCT | RCT | Model analysis | RCT |
| Sample number | CR 99 Control 102 | CR 132 Control 72 | CR 56 Control 57 | ─ | CR 109 Control 95 |
| Target disease | Patients diagnosed with acute myocardial infarction within 6 weeks | Patients with recent acute myocardial infarction or percutaneous coronary intervention | Patients with unstable angina or treated for acute myocardial infarction | acute coronary syndrome (ACS) | CAD patients who suffered from acute coronary syndrome (ACS) |
| Age (years ± SD, intervention/control) | 53 ± 9.5/53 ± 9.5 | 64 ± 11/64 ± 11 | 61 ± 8.7/62 ± 9.4 | ─ | 60 ± 11/62 ± 9 |
| Sex（number of males, rehabilitation/control） | 87/90 | 138/66 | 42/41 | ─ | 80/67 |
| Intervention | Cardiac rehabilitation program (8 weeks) ・Supervised exercise therapy  ・Risk management counseling | CRPP 7 to 14 days  Phase 2:outpatient education and exercise program，twice-weekly・8 weeks  Phase 3:community-based home exercise program，6 months  Phase 4:long-term maintenance，until the end of second years | Start within 2 weeks of leaving hospital, thrice-weekly, 6-week package 60～90 minutes/time (exercise, education, counseling) | Center-based outpatient cardiac rehabilitation | During the first six months, once a week they visited our Cardiac Rehab gym, where they were individually guided in both gym and home-based exercise training by a physical therapist.  Dietary counseling for each patient or a checkup by a medical doctor when appropriate. |
| Control | Usual medical treatment  (includes rehabilitation in community) | Phase 1 only: inpatient ambulating program，7 to 14 days  (2-hour talk with therapist, medication prescription) | Usual medical treatment  (includes rehabilitation in community) | No cardiac rehabilitation | No individually tailored exercise prescriptions. |
| Subgroup analysis | ─ | ・age ・sex ・result of laboratory test | ─ | ・age ・sex  ・clinical presentation  (with or without ACS) | ─ |
| Evaluation period | 1 year | 2 years | 1 year | 1 year | 1 year |
| Setting of hypothesis | ─ | ─ | ─ | ─ | ─ |
| Considered cost 1) CR cost (exercise therapy-relate cost)  2) Additional cost (test, diagnosis and prescription for pre/post CR)  3) Readmission and retreatment cost  4) Cost of other program・additional home care  5) Others (loss in income, etc) | [Cost basis] 1) 4) Staff cost，depreciation cost Other cost (renting space, patient-borne cost)  【Medical fee basis】 2) Cost of investigation | [Cost basis] 1) 4) Staff cost，depreciation cost 2) 3) Other medical cost  Cost of investigation，admission，urgent admission，operation，medication | [Cost basis] 1) 4)Staff cost，material cost  【Medical fee basis】 2) 3) Cost of admission, prescription，clinical examination，investigation: medical care service | [Cost basis]  1)Cost of providing cardiac rehabilitation  3)Cost for the first year after cardiac catheterization  for those who do and those who do not  have a second cardiac event, subsequent  annual cost of care, and the cost of treating  patients who die | [Cost basis] 1) 4)Staff cost，material cost  2) 3) primary health care cost, secondary health care cost, Occupational health care service costs |
| Rationale of cost calculation | ・Rehabilitation cost: statistical data ・Patient’s own expense: estimated by actual condition | ・Cost of rehabilitation, admission: published by hospital ・Cost of drugs: local drug formulary  ・Private practitioner: patient’s self-report | ・DRG ・Schedule of Pharmaceutical Benefits ・Questionnaire to patients and physicians  ・Health system perspectives | ・APPROACH database  ・Total Cardiology Rehabilitation and Risk Reduction Program: salaries,employee benefits, professional development, office supplies, medical supplies, and exercise  equipment, overhead costs ( annual facility, advertising, technology, insurance, and electricity costs) | ・DRG  ・Registries  ・Report of the Social Insurance Institute of Finland |
| Modeling | No difference in survival rate between intervention arm and control arm | Use Kaplan-Meier survival analysis for calculation of mortality | Use Quality Adjusted Survival Analysis (QASA) for calculation of survival years (under investigation) | Markov model | Use Kaplan-Meier survival analysis to examine differences in cumulative major adverse cardiac event |
| Evidence review | ─ | ─ | ─ | ─ | ─ |
| Outcome index | ・QALY ・Mortality ・Work status ・ICUR ・Frequency of health care resource use | ・QALY ・SF-36 ・ICUR | ・QALY ・SF-36 ・ICUR | ・QALY ・ICUR | ・QALY ・15D ・ICUR |
| Method of utility calculation | Time Trade-off  Use mortality rate reported by meta-analysis | Time Trade-off | UBQ-H (Utility Based Quality of life-Heart; disease-specific questionnaire that contains TTO) | EQ-5D-3L | 15D questionnaire (consists of fifteen dimensions: mobility, vision, hearing, breathing, sleeping, eating, speech, elimination, usual activities, mental function, discomfort and symptoms, depression, distress, vitality, and sexual activity) |
| Method of cost-effectiveness analysis | ICUR | ICUR | ICUR | ICUR | ICUR |
| Discount rate of cost | 5% / year | ─ | ─ | 5% / year | ─ |
| Discount rate of outcome | 5% / year | ─ | ─ | 5% / year | ─ |
| Cost of intervention | 480 $ (1 year) ※difference only | 15,291 $ (2 years) | 4,937 $ (1 year) | 45,792.91 $ (1 year) | 2168.73 $ (1 year) |
| Outcome of intervention | 0.071 (QALY, 1 year) ※difference only | 0.6(QALY, survival duration) ※difference only (compared with baseline) | 0.0092886 (QALY, 1 year) ※difference only | 9.77 (QALY, 1 year) | 0.013 (QALY, 1 year) |
| Cost of control | ※difference only | 15,707 $ (2 years) | 4,541 $ (1 year) | 43,179.57 $ (1 year) | 3376.92 $ (1 year) |
| Outcome of control | ※difference only | ※difference only (compared with baseline) | ※difference only | 9.70 (QALY, 1 year) | -0.012 (QALY, 1 year) |
| Result of cost-effectiveness analysis | ICUR:  9,200 ($/ Δ QALY) 1 year 6,800 ($/ Δ QALY) 3 years | SF-36:  Demonstrated improvement in Phase 2 in patients with CRPP  ICUR: - 640 ($/ΔQALY) | ICUR: 42,535 ($/ΔQALY) | The incremental cost per QALY gained ranged from $18,102 for men with ACS older than 75 years to $104,519 for women without ACS younger than 65 years. | Dominant |
| Sensitivity analysis | Performed using the differences in the lower and upper 95%CI limits of utility score | ─ | Performed one-way sensitivity analysis using the differences in the lower and upper 95%CI limits of ICUR and mortality | Performed 1-way sensitivity analysis and probabilistic sensitivity analysis | ─ |
| Conclusion | Cardiac rehabilitation is cost-effective in patients after acute myocardial infarction treatment. | Scores on the SF-36 improved from the early period. and it was highly cost-effective in patients with CRPP. | Rehabilitation showed superiority in improvement of QOL. | Cardiac rehabilitation is most cost effective for those with an ACS and those who are at higher risk for subsequent cardiac events. | Exercise-based cardiac rehabilitation is less costly and more effective than usual care in acute coronary syndrome patients. |
| Generalization | No particular limitation | No particular limitation | No particular limitation | Generalizable to practice | No particular limitation |
| Fiscal impact analysis | ─ | ─ | ─ | ─ | ─ |

RCT, randomized controlled trial

DRG, diagnosis-related group

QALY, quality-adjusted life year

SF-36, 36-Item Short Form Survey

ICUR, incremental cost utility ratio

95%CI, 95% confidence interval

CRPP, Cardiac rehabilitation and prevention program

**Supplementary Material Table 3: Patient characteristics in selected studies by systematic review**

| **Study** | **Item** | **CR arm**  **n (%)** | **UC arm**  **n (%)** |
| --- | --- | --- | --- |
| Oldridge, 1993, Canada | **AMI site** Anterior Inferior Posterior Indefinite | 36 (36) 55 (55)  3 (3)  5 (5) | 34 (33) 56 (55)  8 (8)  5 (5) |
|  | **Previous history** AMI | 17 (17) | 18 (17) |
| Yu, 2004, China | **Indication for CRPP** Myocardial infarction PCI | 129 (71)  52 (29) | 64 (73) 24 (27) |
|  | **Medications** Thrombolytic therapy Antiplatelet drugs β-blockers  Calcium channel blockers Nitrates Statins ACE inhibitors Diuretics  Oral hypoglycemics | 61 (49)  179 (99) 129 (71)  36 (20) 106 (59) 119 (66) 119 (64)  38 (21)  69 (69) | 22 (36) 87 (99) 66 (75)  14 (16) 57 (65) 49 (56) 53 (60) 11 (13) 39 (78) |
|  | **Comorbidities** Smoking Hypertension Hyperlipidemia  Diabetes | 72 (40) 84 (46) 85 (47) 49 (27) | 36 (42) 37 (43) 39 (45) 26 (30) |
| Briffa, 2005, Australia | **Clinical details on index admission** AMI  Unstable angina Thrombolytic therapy PCI/CABG Prior AMI,PCI,CABG Prior CR | 21(37)  36 (63)  8 (14) 34 (60) 21 (37)  3 (5) | 27(48)  29 (52) 14 (25) 26 (46) 28 (50)  3 (5) |
|  | **Medications** Aspirin Antiarrhythmic agent β-blocker ACE inhibitor Calcium channel blockers Long-acting nitrates Diuretic Insulin Hypolipidemic agent  Oral hypoglycemic | 53 (93)  3 (5) 35 (61) 15 (26) 15 (26) 32 (56)  6 (11)  2 (4) 16 (28)  3 (5) | 50 (89)  5 (9) 36 (64) 17 (30) 22 (39) 34 (61)  6 (11)  4 (7) 12 (21)  3 (5) |
|  | **Coronary risk factors**^*^ Family history of coronary artery disease Hypercholesterolemia Hypertension Current smoker Diabetes Obesity | 13 (23) 25 (44) 25 (44) 17 (30)  6 (11)  4 (7) | 13 (23) 26 (46) 29 (52) 20 (36)  9 (16) 12 (23) |
| Hautala, 2017, Finland | **History of AMI**  NSTEMI  STEMI | 47 (48)  44 (45) | 45 (58)  28 (36) |
|  | **Revascularization**  PCI  Earlier CABG | 95 (87)  5 (5) | 83 (87)  8 (8) |
|  | **Medications**  β-blocker ACE inhibitor or ARB  Lipids  Anticoagulants  Calcium channel blockers Nitrates Diuretics | 91 (83)  87 (90)  95 (98)  97 (100)  16 (16)  21 (22)  13 (13) | 83 (87)  67 (86)  77 (99)  77 (99)  20 (26)  22 (28)  17 (22) |

Family history of coronary artery disease: first degree relative aged < 60 years with an acute coronary event

Hypercholesterolemia: total cholesterol level, ≥ 4.5mmol/L

Hypertension: blood pressure, ≥ 140/90 mmHg

Diabetes: fasting plasma glucose level, ≥ 7.8 mmol/L

Obesity: body mass index, > 30 kg·m^-2^

CR, cardiac rehabilitation

CRPP, cardiac rehabilitation and prevention programs

PCI, percutaneous coronary intervention

CABG, coronary artery bypass grafting

NSTEMI, non-ST segment elevation myocardial infarction

STEMI ST, segment elevation myocardial infarction.
